# Zinc alpha 2 glycoprotein as an early biomarker of diabetic nephropathy in patients with type 2 diabetes mellitus

**DOI:** 10.1590/2175-8239-JBN-2018-0200

**Published:** 2019-03-18

**Authors:** Mohamed Elsheikh, Khaled A Elhefnawy, George Emad, Mabrouk Ismail, Maher Borai

**Affiliations:** 1 Faculty of Medicine, Internal Medicine Department, Zagazig University, Egypt.; 2 Faculty of Medicine, Clinical Pathology Department, Zagazig University, Egypt.

**Keywords:** Diabetic Nephropathies, Biomarkers., Nefropatias Diabéticas, Biomarcadores.

## Abstract

**Introduction:**

Although microalbuminuria remains the gold standard for early detection of diabetic nephropathy (DN), it is not a sufficiently accurate predictor of DN risk. Thus, new biomarkers that would help to predict DN risk earlier and possibly prevent the occurrence of end-stage kidney disease are being investigated.

**Objective:**

To investigate the role of zinc-alpha-2-glycoprotein (ZAG) as an early marker of DN in type 2 diabetic (T2DM) patients.

**Methods:**

88 persons were included and classified into 4 groups: Control group (group I), composed of normal healthy volunteers, and three patient groups with type 2 diabetes mellitus divided into: normo-albuminuria group (group II), subdivided into normal eGFR subgroup and increased eGFR subgroup > 120 mL/min/1.73m^2^), microalbuminuria group (group III), and macroalbuminuria group (group IV). All subjects were submitted to urine analysis, blood glucose levels, HbA1c, liver function tests, serum creatinine, uric acid, lipid profile and calculation of eGFR, urinary albumin creatinine ratio (UACR), and measurement of urinary and serum ZAG.

**Results:**

The levels of serum and urine ZAG were higher in patients with T2DM compared to control subjects and a statistically significant difference among studied groups regarding serum and urinary ZAG was found. Urine ZAG levels were positively correlated with UACR. Both ZAG levels were negatively correlated with eGFR. Urine ZAG levels in the eGFR ˃ 120 mL/min/1.73m^2^ subgroup were higher than that in the normal eGFR subgroup.

**Conclusion:**

These findings suggest that urine and serum ZAG might be useful as early biomarkers for detection of DN in T2DM patients, detectable earlier than microalbuminuria.

## Resumo

**Introdução:**

Embora a microalbuminúria continue sendo o padrão ouro para a detecção precoce da nefropatia diabética (ND), ela não é um preditor suficientemente preciso do risco de ND. Assim, novos biomarcadores para prever mais precocemente o risco de ND e possivelmente evitar a ocorrência de doença renal terminal estão sendo investigados.

**Objetivo:**

Investigar a zinco-alfa2-glicoproteína (ZAG) como marcador precoce de ND em pacientes com debates mellitus tipo 2 (DM2).

**Métodos:**

Os 88 indivíduos incluídos foram divididos em quatro grupos: grupo controle (Grupo I), composto por voluntários saudáveis normais; e três grupos de pacientes com DM2 assim divididos: grupo normoalbuminúria (Grupo II), subdivididos em TFG normal e TFG > 120 mL/min/1,73 m^2^), grupo microalbuminúria (Grupo III) e grupo macroalbuminúria (Grupo IV). Todos foram submetidos a urinálise e exames para determinar glicemia, HbA1c, função hepática, creatinina sérica, ácido úrico, perfil lipídico, cálculo da TFG, relação albumina/creatinina (RAC) e dosagem urinária e sérica de ZAG.

**Resultados:**

Os níveis séricos e urinários de ZAG foram mais elevados nos pacientes com DM2 em comparação aos controles. Foi identificada diferença estatisticamente significativa entre os grupos estudados em relação aos níveis séricos e urinários de ZAG. Os níveis urinários de ZAG foram positivamente correlacionados com a RAC. Ambos os níveis de ZAG foram negativamente correlacionados com TFG. Os níveis urinários de ZAG no subgrupo com TFG ˃ 120 mL/min/1,73m^2^ foram maiores do que no subgrupo com TFG normal.

**Conclusão:**

Constatamos que a ZAG sérica e urinária pode ser um útil biomarcador precoce para detecção de ND em pacientes com DM2, sendo detectável mais precocemente que microalbuminúria.

## Introduction

Diabetic nephropathy is associated with mortality and morbidity in patients with diabetes mellitus.^1^ The most common method of detecting the early signs of diabetic nephropathy is the measurement of microalbuminuria.^2^ However, pathological abnormalities have been reported to occur before the onset of microalbuminuria.^3^ In chronic cases of diabetic nephropathy, renal function correlates better with the degree of tubulointerstitial injury rather than with glomerular lesions, suggesting that researchers should look for tubular biomarkers in order to identify patients with diabetic nephropathy.^4^ There has been an increasing interest in identifying other biomarkers that might give a sensitive and rapid means of detecting the progression of diabetic nephropathy. In this aspect, biomarkers that reflect tubular damage have been suggested by many investigators.^5^^,^ 6

Zinc-alpha-2-glycoprotein (ZAG) is a protein of interest because of its ability to play many important functions in the human body, including fertilization and lipid mobilization. Its structural organization and folding characteristics are similar to the MHC class I antigen-presenting molecule; hence, ZAG may have a role in the immune response. The function of ZAG under physiologic and cancerous conditions remains mysterious; however, it is considered a tumor biomarker for various carcinomas. There are several unrelated functions attributed to ZAG, such as RNase activity, regulation of melanin production, hindering of tumor proliferation, and transport of nephritic by-products^7^.

ZAG is present in a variety of epithelia and is secreted into many body fluids.^8^ It was found that urine ZAG increased specifically in patients with diabetes and it may be used as a biomarker for specific and accurate analysis of diabetic nephropathy.^9^ Immunohistochemical analyses have shown that ZAG is expressed mainly in the tubules of the human kidney.^10^

We hypothesized that the urine and serum concentrations of ZAG might increase earlier than microalbuminuria in diabetic nephropathy. This study aimed to determine the role of ZAG in the early diagnosis of diabetic nephropathy by estimating the concentrations of urine and serum ZAG in patients with type 2 diabetes mellitus (T2DM), according to their levels of albuminuria.

## Subjects and methods

### Study design:

This was a case-control study carried out in the Internal Medicine and Clinical Pathology departments, Faculty of Medicine, Zagazig University, from December 2017 to August 2018.

### Participants and groups:

A total of 88 persons were included after their written informed consent and classified into 4 main groups. Control group (group I) were normal healthy volunteers (n = 22). The three T2DM patient groups were divided according to urinary albumin/creatinine ratio (UACR) into: normo-albuminuria group (group II) (UACR < 30 mg/g, n = 22, further subdivided according to eGFR into 2 subgroups: normal eGFR subgroup and increased eGFR subgroup > 120mL/min/1.73m^2^), DN group with microalbuminuria (group III, UACR from 30 to 300 mg/g, n = 22) and DN with macroalbuminuria (group IV, UACR > 300 mg/g, n = 22). All groups were matched for age, sex, and body mass index. 

### Exclusion criteria:

Patients with hepatic diseases, heart failure, thyroid disorders, autoimmune diseases, inflammatory conditions and sepsis, malignancy, renal impairment of known origin, urinary tract infections, past history of rapidly progressive renal failure, any type of glomerulonephritis, and patient with polycystic kidney were excluded from the study.

### Physical examination and measurements:

All subjects in the study were subjected to A) assessment of medical history and thorough clinical examination according to patients' records; B) routine investigations according to the methods applied in the clinical pathology laboratories of Zagazig University hospitals including urine analysis, complete blood count, fasting and random blood glucose levels, HbA1c, liver function tests, serum creatinine, urea, uric acid, lipid profile and calculation of eGFR. The modification of diet in renal disease (MDRD) equation was used for eGFR (mL/min/1.73 m^2^): 175 x (Scr)^-1.154^ x (Age)^-0.203^ x (0.742 if female)^11^. The urine albumin was divided by 100 to convert mg/dL to g/L and then the urine albumin value was divided by the urine creatinine value to albumin creatinine ratio (UACR) (mg/g) (urine albumin (mg/L) x100/ urine creatinine (mg/dL)). UACR was reported as (mg albumin/g creatinine)^12^. C) Specific investigations included measurement of urinary and serum ZAG by human ZAGp1 (Zinc-alpha-2-glycoprotein) ELISA Kit (Spanbiotec, Guandong, China). D) Other investigations included electrocardiogram and abdominal ultrasound.

### Statistical analysis

The collected data were computerized and statistically analyzed using SPSS program (Statistical Package for Social Science) version 18.0. Qualitative data were presented as frequencies and relative percentages. Chi-square test was used to calculate difference between qualitative variables. Quantitative data were presented as mean ± SD (standard deviation). Independent T-test was used to compare differences between quantitative variables in two groups with normally distributed data. ANOVA F-test test was used to compare differences between quantitative variables in more than two groups with normally distributed data. Kruskal Wallis test was used to compare differences between quantitative variables in more than two groups with non-normally distributed data. Pearson correlation coefficient was used to calculate correlation between quantitative variables. Receiver operating characteristic (ROC) curve analysis was used to identify optimal cut-off values of Vnn-1 with maximum sensitivity and specificity for prediction of the disease. Accuracy was measured by the area under the ROC curve. *p*-values > 0.05 indicated non-significant results, < 0.05 indicated significant results and < 0.01 indicated highly significant results.

## Results

**Figure 1:**
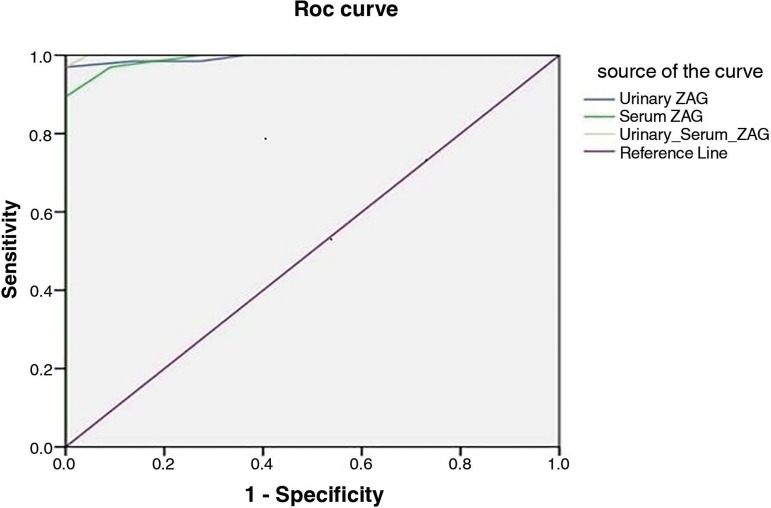
Roc curve for Validity of urinary and serum ZAG in prediction of albuminuria.

Our results showed that there was no significant difference among groups regarding age, weight, and sex while there was a significant difference regarding the duration of diabetes mellitus (Table 1). We found significant differences among groups regarding fasting blood glucose (FBG), random blood glucose (RBG), HbA1C, serum creatinine, eGFR, urinary albumin/creatinine ratio, serum albumin, and total plasma proteins (Table 2). There were significant differences among groups regarding both urinary and serum ZAG and estimated GFR (Table 3). Table 3 shows the number and percentage of subjects according to eGFR in all groups. There was a significant positive correlation between both urinary and serum ZAG and duration of DM, UACR, and with each other, while a negative significant correlation was found between both urinary and serum ZAG and serum albumin, total plasma proteins, and eGFR (Table 4). There was a significant increase in serum and urinary ZAG in cases with eGFR > 120 mL/min when compared to cases with normal eGFR (90 to 120 mL/min) in the normoalbuminuric group II (Table 5). The accuracy of urinary ZAG was 95.5%, and that of serum ZAG was 90.9%; considering both of them, the accuracy was 95.5% (Table 6 & Figure 1).

**Table 1:** Comparison of different variables among the study groups

Variable	Group I (n = 22)		Group II (n = 22)		Group III (n = 22)		Group IV (n = 22)		F	*p*
Age (years) Mean ± SD	51 ± 6.62		51 ± 6.91		51 ± 6.29		51 ± 5.76		0.01	0.99
Weight (Kg): Mean ± SD	80.36 ± 7.65		79.59 ± 9.42		81.18 ± 9.12		80.95 ± 6.59		0.16	0.92
DM Duration (years) Mean ± SD	-------		4.59 ± 0.95		7.91 ± 0.97		12.68 ± 2.82		111.62	< 0.001
Variable	No	%	No	%	No	%	No	%	χ2	*p*
Sex										
Male	13	59.1	11	50	12	54.5	13	59.1	0.51	0.92
Female	9	40.9	11	50	10	45.5	9	40.9		

*p*-value < 0.05 is significant. DM: diabetes mellitus, SD: standard deviation

**Table 2:** Comparison of different variables among the study groups

Variable	Group I (n = 22)	Group II (n = 22)	Group III (n = 22)	Group IV (n = 22)	F	*p*
FBG: (mg/dl) Mean ± SD	79.89 ± 13.51	131.36 ± 56.65	141.82 ± 73.37	150.89 ± 94.83	6.354	0.001
RBG: (mg/dl) Mean ± SD	92.07 ± 8.23	160.93 ± 93.86	194.64 ± 98.85	180.14 ±66.26	10.020	< 0.001
HbA1c: (%) Mean ± SD	5.19 ± 0.335	7.77 ± 1.74	8.64 ± 1.49	8.49 ± 1.18	18.428	< 0.001
S. uric acid: (mg/dl) Mean ± SD	5.28 ± 1.09	4.63 ± 1.03	4.71 ± 1.07	4.97 ± 1.17	1.59	0.2
S. Cr: (mg/dL) Mean ± SD	0.882 ± 0.136	1.13 ± 0.533	3.48 ± 1.16	5.45 ± 2.91	45.749	< 0.001
eGFR: (mL/min) Mean ± SD	108.73 ± 7.11	124.09±11.23	98.86 ± 3.85	82.36 ± 4.87	125.43	< 0.001
UACR: (mg/g) Mean ± SD	20.4 ± 5.11	20.72 ± 5.37	77.74 ± 28.93	383.55 ± 61.59	569.05	< 0.001
Albumin: (g/dL) Mean ± SD	4.53 ± 0.66	4.15 ± 0.54	4.19 ± 0.58	3.31 ± 0.08	22.06	< 0.001
T. protein: (g/dL) Mean ± SD	7.26 ± 0.55	7.12 ± 0.52	7.21 ± 0.54	6.39 ± 0.09	16.85	< 0.001

FBG: fasting blood glucose, RBG: random blood glucose, HbA1C: glcosylated hemoglobin, S. Cr: serum creatinine, eGFR: estimated glomerular filtration rate, UACR: urinary albumin creatinine ratio, *p*-value < 0.05 is significant.

**Table 3:** Comparison of eGFR, and urinary and serum ZAG among the studied groups

Variable	Group I (n = 22)		Group II (n = 22)		Group III (n = 22)		Group IV (n = 22)		F	*p*
Urinary ZAG: (mg/g) Mean ± SD	26.91 ± 2.41		36.86 ± 3.76		46.09 ± 2.31		56.73 ± 2.62		444.93	< 0.001
Serum ZAG: (mg/l) Mean ± SD	20.27 ± 1.52		24.55 ± 1.68		32.23 ± 2.11		40.82 ± 1.89		545.43	< 0.001
eGFR:	No	%	No	%	No	%	No	%	χ2	*p*
< 90	0	0	0	0	0	0	19	86.4		
90 - 120	22	100	8	36.4	22	100	3	13.6	119.7	< 0.001
> 120	0	0	14	63.6	0	0	0	0		

ZAG: zinc-alpha-2-glycoprotein, eGFR: estimated glomerular filtration rate, *p*-value < 0.05 is significant.

**Table 4:** Correlation between both urinary and serum ZAG and different variables among the three patients groups

Variable	Urinary ZAG (n = 66)		Serum ZAG (n = 66)	
	r	*p*	r	*p*
Age (years)	0.06	0.63	0.002	0.98
Weight	0.13	0.31	0.07	0.56
Duration of DM (years)	0.88	< 0.001	0.86	< 0.001
Albumin (g/dL)	-0.51	< 0.001	-0.52	< 0.001
Total protein (g/dL)	-0.49	< 0.001	-0.47	< 0.001
FBS: (mg/dL)	0.04	0.74	0.03	0.84
RBG: (mg/dL)	-0.02	0.9	-0.02	0.99
HbA1c: (%)	-0.08	0.51	-0.02	0.86
eGFR: (mL/min)	-0.78	< 0.001	-0.87	< 0.001
Uric acid: (mg/dL)	0.16	0.2	0.12	0.34
Creatinine:(mg/dL)	0.15	0.23	0.08	0.46
UACR: (mg/g)	0.86	< 0.001	0.89	< 0.001
Urinary ZAG: (mg/g)	-----	------	0.93	< 0.001
Serum ZAG: (mg/L)	0.93	< 0.001	------	-----

DM: diabetes mellitus, FBG: fasting blood glucose, RBG: random blood glucose, HbA1C: glycosylated hemoglobin, S.Cr: serum creatinine, eGFR: estimated glomerular filtration rate, UACR: urinary albumin creatinine ratio, ZAG: zinc-alpha-2-glycoprotein, *p*-value < 0.05 is significant.

**Table 5:** Relation between urinary and serum ZAG and eGFR in Group II and Group IV

Variable	Group II		t	*p*
	GFR 90 - 120 (n = 8)	GFR > 120 (n = 14)		
Urinary ZAG: (mg/g) Mean ± SD	32.63 ± 2.77	39.29 ± 1.14	7.99	< 0.001
Serum ZAG: (mg/L) Mean ± SD	24.38 ± 1.51	26.44 ± 1.82	2.71	0.01
Variable	Group IV		t	*p*
	GFR < 90 (n = 19)	GFR 90 - 120 (n = 3)		
Urinary ZAG: (mg/g) Mean ± SD	56.63 ± 2.69	57.33 ± 2.52	0.42	0.78
Serum ZAG: (mg/L) Mean ± SD	40.37 ± 1.61	41.67 ± 0.58	1.36	0.19

ZAG: zinc alpha 2 glycoprotein, *p*-value < 0.05 is significant

**Table 6:** Validity of Urinary and serum ZAG in prediction of albuminuria

Variable	Cutoff	AUC	Sens.	Spec.	+PV	-PV	Accuracy	*p*-value
Urinary ZAG	≥ 28.5	0.99	98.5	86.4	95.6	95	95.5	< 0.001
Serum ZAG	≥ 22.5	0.99	97	72.7	91.4	88.9	90.9	< 0.001
Both	--	0.99	97	90.9	96.9	90.9	95.5	< 0.001

ZAG: zinc alpha 2 glycoprotein, NPV: negative predictive value, PPV: positive predictive value, *p*-value < 0.05 is significant.

## Discussion

Microalbuminuria is considered the earliest clinical manifestation of DN.^3^ DN affects all cellular components in the glomeruli and renal tubular interstitium.^4^ As glomerular damage usually results in proteinuria, much research has been undertaken on glomerular damage in patients with T2DM.^13^ However, some patients with diabetes can have a decrease in eGFR and may progress to end-stage renal disease without having any significant albuminuria.^14^ Some patients with microalbuminuria have advanced renal pathological changes for which therapy is less effective than one might usually expect for those with early stage disease.^13^^,^ 14 The correlation between albuminuria and eGFR has been found to be weak and urinary albumin lacks both sensitivity and specificity to detect early stages of DN.^14^

Several tubular biomarkers that can predict renal damage in patients with early diabetic nephropathy have been investigated, such as neutrophil-gelatinase associated lipocalin, kidney injury molecule 1, and liver fatty acid binding protein.^7^

Our study aimed to investigate the role of ZAG in early diagnosis of DN by estimating the concentrations of urine and serum ZAG in patients with T2DM, according to their levels of albuminuria.

Because our groups were matched regarding age, body weight and sex, the effect of these factors on the results of urinary and serum ZAG in our study were excluded. A significant difference was found regarding the duration of T2DM which is in agreement with other studies that found a significant difference between normo- and microalbuminuric groups regarding duration of diabetes mellitus.^15^^-^ 17

The results for FBG, RBG, HbA1C, serum creatinine, eGFR, urinary albumin/creatinine ratio, serum albumin and total plasma proteins were in agreement with another study, which documented a significant difference between both diabetic groups regarding the previous parameters.^18^ Another study reported that progression of diabetic nephropathy was accompanied by declining GFR and increasing urinary albumin excretion.^19^ We found that serum albumin in Group IV was statistically decreased compared to the other groups, which is in line with another study that found that serum albumin was significantly lower in the macroalbuminuric group of DN patients^20^.

A significant difference between control and normo-albuminuric groups was found as for GFR by MDRD. These results were demonstrated by other studies^21^^-^ 23. This can be explained by the pathogenesis of diabetic nephropathy, as there is hyperfiltration in stage 1 due to an imbalance in afferent and efferent arteriolar resistance, resulting in increased glomerular hydrostatic pressure and hyperfiltration. Activation of the renin-angiotensin system (RAS) increases angiotensin II levels, leading to efferent arteriolar vasoconstriction and production of proinflammatory and profibrotic molecules through multiple mechanisms.^24^

Regarding GFR, other studies also found significant difference between control and normo-albuminuric patients (high eGFR subgroup), being lower in the normoalbuminuric group.^25^^,^ 26 On the other hand, our study found significant differences regarding GFR by MDRD between control and micro-albuminuric groups, which is in line with another study that reported that albuminuria is the strongest risk factor for fast annual eGFR decline.^27^

In our study, a significance differences was found among studied groups in both urinary and serum ZAG levels. The increase of urinary ZAG in diabetic groups is the result of the proximal tubules in the kidneys being particularly susceptible to diabetes-associated injury, as they are subjected to prolonged exposure to various metabolic and hemodynamic disturbances.^28^ In prolonged cases of DN, renal function correlates better with the degree of tubulointerstitial injury than the degree of glomerular lesions.^29^

As ZAG is mainly expressed in the proximal convoluted and straight tubules,^10^ the changes in ZAG urine concentrations observed in our study might be indicative of the tubular damage that is present in earlier stages of diabetic nephropathy, preceding those that result in microalbuminuria.

Another study reported that the concentration of ZAG in urine was higher than that in serum, especially in patients with T2DM, which suggests that the increased urine concentrations of ZAG were mainly due to increased ZAG secretion by tubular epithelial cells.^30^ Other studies demonstrated that urine ZAG levels were progressively increased in diabetic patients with normo-, micro-, and macroalbuminuria indicating that it is positively related with diabetes nephropathy progression.^31^

Moreover, other studies indicated that the appearance of ZAG in albumin-negative urine samples preceded the appearance of albumin in T2DM patients from South Asia, suggesting that ZAG may be an early novel urinary biomarker useful for the screening of non-albuminuric DN.^32^

In our study there were significant positive correlations between both urinary and serum ZAG and duration of DM, UACR, and with each other. Negative significant correlation was found between both urinary and serum ZAG and albumin, total plasma proteins and eGFR, in contrast with another study that reported an inverse relationship between ZAG levels and plasma proteins.^33^

We found that urine concentrations of ZAG were significantly increased in patients with T2DM with higher eGFR compared with T2DM patients with normal eGFR.

The serum ZAG concentration was positively correlated with eGFR but not with glucose levels, body weight and serum creatinine, which is in agreement with another study.^30^

## Conclusion

The strong positive association between urinary ZAG concentrations and UACR, and the earlier appearance of urine ZAG compared with albuminuria, suggest that ZAG might be a useful biomarker for the early diagnosis of DN in patients with T2DM.

Large-scale prospective studies are needed to comprehensively understand the potential pathophysiological role of ZAG in DN and to determine the cause-effect relationship between urine and serum concentrations of ZAG and DN.
