# Erratum: Zinc alpha 2 glycoprotein as an early biomarker of diabetic nephropathy in patients with type 2 diabetes mellitus

**DOI:** 10.1590/2175-8239-JBN-2018-0200er

**Published:** 2019-08-01

In the article “Zinc alpha 2 glycoprotein as an early biomarker of diabetic nephropathy in patients with type 2 diabetes mellitus”, with DOI code http://dx.doi.org/10.1590/2175-8239-jbn-2018-0200, published in the Brazilian Journal of Nephrology, ePub ahead of print on March 18, 2019, the name and information of the first author was missing:

In the original version the information was:

Khaled A Elhefnawy^1^ http://orcid.org/0000-0002-2024-5449

George Emad^1^

Mabrouk Ismail^1^

Maher Borai^2^

^1^Faculty of Medicine, Internal Medicine Department, Zagazig University, Egypt.

^2^Faculty of Medicine, Clinical Pathology Department, Zagazig University, Egypt.

The correct information is:

Mohamed Elsheikh^1^

Khaled A Elhefnawy^1^ http://orcid.org/0000-0002-2024-5449

George Emad^1^

Mabrouk Ismail^1^

Maher Borai^2^

^1^Faculty of Medicine, Internal Medicine Department, Zagazig University, Egypt.

^2^Faculty of Medicine, Clinical Pathology Department, Zagazig University, Egypt.

